# Stem Cells and Regenerative Medicine: Myth or Reality of the 21th Century

**DOI:** 10.1155/2015/734731

**Published:** 2015-08-02

**Authors:** J.-F. Stoltz, N. de Isla, Y. P. Li, D. Bensoussan, L. Zhang, C. Huselstein, Y. Chen, V. Decot, J. Magdalou, N. Li, L. Reppel, Y. He

**Affiliations:** ^1^CNRS, UMR 7365, Lorraine University, 54500 Vandoeuvre, France; ^2^Nancy Hospital (CHU), Cell and Tissue Therapy Unit (UTCT), 54500 Vandoeuvre, France; ^3^Lorraine University, 54000 Nancy, France; ^4^Medical College and Zhongnan Hospital, Wuhan University, Wuhan, China; ^5^Service de Thérapie Cellulaire, Calmette Hospital, Kunming, China; ^6^Medical School, Wuhan University, Wuhan, Hubei, China; ^7^Anzhen Hospital, Cardiovascular and Lung Research Center, Beijing, China

## Abstract

Since the 1960s and the therapeutic use of hematopoietic stem cells of bone marrow origin, there has been an increasing interest in the study of undifferentiated progenitors that have the ability to proliferate and differentiate into various tissues. Stem cells (SC) with different potency can be isolated and characterised. Despite the promise of embryonic stem cells, in many cases, adult or even fetal stem cells provide a more interesting approach for clinical applications. It is undeniable that mesenchymal stem cells (MSC) from bone marrow, adipose tissue, or Wharton's Jelly are of potential interest for clinical applications in regenerative medicine because they are easily available without ethical problems for their uses. During the last 10 years, these multipotent cells have generated considerable interest and have particularly been shown to escape to allogeneic immune response and be capable of immunomodulatory activity. These properties may be of a great interest for regenerative medicine. Different clinical applications are under study (cardiac insufficiency, atherosclerosis, stroke, bone and cartilage deterioration, diabetes, urology, liver, ophthalmology, and organ's reconstruction). This review focuses mainly on tissue and organ regeneration using SC and in particular MSC.

## 1. Introduction

Most of human tissues and organs do not regenerate spontaneously, justifying why cell therapy is today a significant tissue and organ repair strategy. The concept of regenerative medicine is an emerging multidisciplinary field to revolutionize the way “*to improve the health and quality of life by restoring, maintaining or enhancing tissue and functions of organs.*”

The history of SC began in the mid nineteenth century with the discovery that some cells could generate other cells. In the beginning of the 20th century, SC were discovered when it was found that the bone marrow contained hematopoietic SC and stromal cells [1, 2]. The first successful transplant was performed by Dr. Thomas in Cooperstown, NY, in the late 1950s. The transplant involved identical twins, one of whom had leukemia, avoiding the problems associated with nontwin transplants, such as graft-versus-host disease [3]. It was not until 1968 that the first successful nontwin (allogeneic) transplant was performed. In this case, the donor was a sibling of the patient [4]. The first successful unrelated donor transplant took place in 1973 in New York when a young boy with a genetic immunodeficiency disorder received multiple marrow transplants from a donor identified as a match through a blood bank in Denmark. The first successful unrelated donor transplant for a patient with leukemia took place in 1979 at the Hutchinson Center. Since then, bone marrow transplantation expanded rapidly during the 1990s [5].

In 1998, cells from the inner cell mass of early embryos were isolated as the first embryonic stem cell lines [6, 7]. Later, in 2006, Takahashi et al. described the IPS (induced pluripotent stem cells) [8–10]. Several categories of stem cells can be used in regenerative medicine including embryonic stem cells (ESC), fetal stem cells (FSC), and adult stem cells (ASC) [11, 12]. Not all stem cells are of equal interest in terms of ability for clinical applications and are able to evolve into different specialized cells. Fetal and adult stem cells are undifferentiated cells, which can be found within fetus or in adult tissues or organs. They are able of limited self-renewal and are multipotent, which means that they can differentiate in several types of tissue cells. Although adult stem cells cannot be expanded in culture indefinitely, the use of these cells does not present ethical problems.

Multipotent SC, self-renewing, and adherent MSC, represent a small fraction of the marrow stroma [13–21]. These nonhematopoietic stromal cells are usually harvested* in vitro* from bone marrow but also from other tissues of mesodermal origin: fetal or neonatal tissues (umbilical cords or placenta), adipose tissue, joint synovium, dental pulp, and so forth [22–30]. MSC are characterized by their capacity of self-renewal and differentiation in different cells types (chondrocytes, endothelial cells,…). They were initially identified as progenitors able to produce colonies of fibroblast-like cells (CFU-F for colony forming units-fibroblast), to differentiate into bone or cartilaginous tissues, and to support hematopoiesis. Indeed, MSC cultivated under adapted conditions differentiate into cells of conjunctive tissues: osteoblasts, chondrocytes, tenocytes, adipocytes, and stromal cells supporting the hematopoiesis [31]. They can also differentiate into vascular smooth muscle cells, sarcomere muscular cells (skeletal and cardiac), and endothelial cells [32–36]. Recent publications even state that they can differentiate into nonmesodermal cells such as hepatocytes, neurons, or astrocytes [37–42].

MSC do not have a defined profile of surface antigen expression but there are available markers to identify them. They are mainly characterized by the expression of different antigens, CD105, CD73, CD90, Stro-1, CD49a, CD29, and CD166. On the other hand, MSC do not express antigens CD34 and CD45 (specific of the cells of hematopoietic origin), glycophorin (specific of blood cells), antigens of differentiation of the various leucocyte populations (CD14, CD33, CD3, and CD19), and HLA-DR [43–46]. The International Society for Cellular Therapy suggested a consensual definition: cells must adhere on plastic, express CD75, CD90, and CD105 and not CD34, CD45, HLA-DR, or CD11b, CD19, and are capable of differentiation into chondrocytes, osteoblasts, and adipocytes [26, 47]. Under current conditions of* in vitro* culture [48], the results obtained showed that the proliferation of MSC remained within the limit of Hayflick of 40* in vitro* population doublings but was affected by the age of the donors [49–54]. Recent studies show that the ability of expansion and differentiation of MSC is donor-dependent. It seems that the number of MSC and their ability of* in vitro *differentiation and tissue regeneration* in vivo* decrease with age and according to the donor pathology [55]. They generally do not circulate in the peripheral blood but are resident in mesenchymal tissues [56]. Bone marrow mesenchymal stem cells (BM-MSC) can provide a support for the growth of the hematopoietic stem cells through the secretion of cytokines and through the creation of cellular interactions either directly (adhesion molecules) or indirectly (production of the extracellular matrix components). Today, nonstandardized protocols exist for their culture, differentiation, and self-renewal ability. In addition, some MSC could be more immature, without any tissue specialization, and their existence has been suspected in human [57–59].

IPS result in the acquisition of a novel state followed by the* in vitro* reprogramming of an adult cell after addition of selected transcription factors. The major advance in this field was performed in 2006 with the possibility of a direct reprogramming of somatic cells into pluripotent cells starting from fibroblasts [8, 9]. Generation of IPS depends on the genes used for the induction (*Oct 3-4* and* Sox* gene family are determinant regulators for the induction process). In the course of the reprogramming, an extinction of the characteristic genes of the fibroblast, a reexpression of embryonic genes (*SSEA 1* and* 4*), and activation of telomerase are observed. However, the efficiency of the technique is low. It is likewise necessary to underline that the IPS are exposed to a significant risk of malignant transformation due to the presence of the oncogene c-Myc used in the reprogramming. The present interest of this type of lines and its nonembryonic origin is the possibility of establishing specific lines of deficient patients for clinical research. The IPS are thus a tool for the study of the mechanisms of cell differentiation and genetic diseases and also for pharmacological screening [60].

## 2. Main Clinical Applications of Stem Cells

The majority of medicine specialities and different applications can benefit in the next decade from the progress in regenerative medicine: most are at experimental stages, with the exception of bone marrow transplantation. Cell therapy covers very large potentials in many clinical fields in cancer and in regenerative medicine [61–63], and more than 3,000 trials with SC are currently in progress (https://www.clinicaltrials.gov/).

Nevertheless, before SC therapeutics can be applied in the clinic, more research is necessary to understand their behaviour upon transplantation as well as the mechanisms of their interaction with the diseased microenvironment. Many authors underlined that regenerative medicine is likely to transform in the future the way we practice medicine, using pharmacological or surgical procedures. The mechanism of action of SC is still being determined. The general consensus today suggests that the most probable mechanism may be through the release of cytokines and other growth-promoting factors.

Before clinical applications, many challenges are to be solved [64].

- How to differentiate SC to the desired cell phenotype and which biological and environmental parameters are important during culture for differentiation?
- What are the best suitable cells: which precursors or differentiated cells?
- What are the possible immunological barriers when allogenic cells are used?
- What are the best biomarkers to identify pluripotent/multipotent/precursors cells?
- What is the role of the microenvironment (scaffolds, mechanical signals)? [65]
- What are the bioreactive molecules such as cytokines or growth factors that can support the formation of the desired tissue?
- Are there potential karyotype changes during cell culture?
- The translation from laboratory to clinics by using good laboratory practice (GPL) could impact on cell properties?
- Which are the best methods to trace cells* in vivo*?

It is important to note that clinical applications of biotherapies are strongly controlled in Western countries. Harvesting cells or tissues of human origin can only be performed in health centers accredited by Public Authorities (in France, different regulation laws describe the procedure of authorization related to preparation, storage, and clinical use of cells and tissues). The European Regulating Authorities are also very strict about the nature of the clinical trials and about the choice of the patients. Before grafting, different points must be precised.

- The severity degree of the pathology has to be considered.
- What type of grafting is planned for the patients?
- The site of grafting should be defined.
- What is the benefit for the patient?
- What is the clinical evaluation method to investigate the functionality of the graft?
- Possible side effects.

### 2.1. Stem Cells and Cancer

Cancer SC has been for long a concept of hematology, particularly in acute myeloid leukaemia. However, more recently, research studies have described the concept of tumor initiating cells in solid tumors [66–72]. The anticancer cell therapy includes bone marrow grafting and in particular the injection of autologous or allogenic hematopoietic stem cells (HSC) CD34+. This population (CD34+) is however heterogeneous regarding its ability to generate the various lines and is the object of many research studies [73]. The graft of HSC has gained an essential place in therapeutic oncohematology [74, 75]. By 1950s, the fundamental role of hematopoietic tissue in protection against radiations was highlighted. The first clinical trials in 1959 showed the feasibility of an engraftment of allogenic marrow [3]. In 1968, the first compatible allogenic grafts HLA were successfully carried out among patients presenting severe combined deficits [4]. Then, the first cryopreserved autografts of bone marrow were reported in lymphomas. Since then, studies were pursued to improve the clinical trials and to decrease, in autologous situations, the relapses linked to the residual disease often present in the graft. Other studies aimed to prevent, in allogenic situations, the graft versus host disease [76].

Using chemical agents or specific monoclonal antibodies,* ex vivo* manipulations of grafts were developed to eliminate tumoral cells or T lymphocytes. By 1984, new sources of HSC have been highlighted in the peripheral and placental blood [77, 78]. That is a major step toward the development of grafts of blood HSC. The first placental blood graft was performed by Gluckman in Paris in 1998 [79]. Since 1993, banks of cryopreserved grafts of placental origin have been developed [80–82].

The use of cytotoxic T cells or NK cells, isolated and amplified* in vitro*, can be proposed for anticancer applications [83–87]. The use of B cells, CD4+ T cells, regulatory T cells [88, 89], and myeloid dendritic or predendritic cells producing interferon is also possible. The injection of dendritic cells for antitumoral immunization, mainly in the residual disease, but also as adjuvant therapy, is the basis of different clinical trials. However, much remains to be understood as the cells nature, their capacity to homing to specific sites (tumor, nodes), and their capacity to stimulate the immune system.

Several clinical trials have been proposed (34 in the beginning of 2014). The main applications are MSC and graft failure, graft versus host disease, and treatment of myelodysplasia [90, 91]. This point will not be developed in this review but a lot of information can be found in the literature [92–94].

### 2.2. Stem Cells and Tissue Regeneration

Regenerative medicine, based on the graft of tissue native cells (i.e., myocytes, chondrocytes, etc.) or SC able to differentiate into somatic cells, holds great promise if clinical hurdles can be overcome, particularly their possible tumorigenic property. This was highlighted in a case report involving a child who received fetal neural SC as a treatment for a neurodegenerative disease, but who later unfortunately developed multifocal glioneuronal tumor from transplanted neural stem cells [95]. Many studies have been published in this area in the last 20 years [96–100].

The regeneration of damaged tissues or organs implies the existence of cells able to proliferate, differentiate, and give a functional contribution to the regenerative processes. Among the possible middle-term therapeutic applications, cardiac insufficiency, atherosclerosis, osteoarticular diseases, diabetes, and liver diseases can be considered.

In regenerative medicine, four important issues have to be taken into account: (1) the choice of the reparative cells that can form a functional tissue; (2) if necessary, the choice of appropriate scaffolds for transplantation; (3) the role of bioreactive molecules, such as cytokines and growth factors that support the formation of the desired tissue; (4) grafting and safety studies (GMP compliance). More than 3,000 clinical trials are indexed in “https://www.clinicaltrials.gov/” (mainly in USA (25%), Europe (30%), and Asia (40%)), with most of them using MSC.

#### 2.2.1. Heart Disease

Every year in France, 10,000 new cases of serious cardiac insufficiency are detected. Heart transplants remain the only treatment for the most advanced stages but the shortage of donors and complications of immunosuppression restrict the indications. Surgical remodeling of the left ventricle only deals with the particular anatomical forms and recent negative results have led to a review of the indications. Mechanical ventricular assistance remains a temporary solution for those waiting for a transplant. There is thus a need for new treatment solutions. Xenotransplantation is not progressing since the immunological challenges are considerable and there are major safety considerations. Gene therapy and IPS are still in their infancy [27, 101] and the complexity of the mechanisms involved in heart failure does not lend itself to this therapeutic approach. Finally, cell therapy has a place, but only in patients who retain a sufficient reserve of contractile cells. The numerous trials have not made it possible to reach a conclusion at the present time [102–109].

Today more than 40 clinical trials are listed with a majority of bone marrow, Wharton's jelly and adipose stem cells [110–113]. Histologic observations in autopsy of samples of allogenic cardiac grafts in sex mismatch showed the formation of cardiomyocytes with the receiver genotype in the myocardial tissue coming from the donor [50]. Y genotype cardiomyocytes have been shown in the myocardium of female mice that received an intravenous injection of bone marrow coming from male mice. Isotypic studies showed the homing of progenitor stem cells from bone marrow towards the lesion sites after a coronary ligation. The molecular signals leading to tissue repair are unknown. However some cytokines released during cardiac ischemia could be involved.

The treatment of myocardial infarction (MI), however, is subject to a significant constraint: the immediate availability of cells. The intracoronary injection of stem cells prepared starting from a withdrawal of bone marrow did not lead to significant improvements (3% maximum of the ejection fraction of the left ventricle). In the same manner, the intravenous injection of MSC does not give significant results. In the case of heart failure, the cell therapy turns out to be no efficient and it seems difficult today to envisage a regenerative therapy. At the end of 2007, the US based stem cell company* Osiris Therapeutics* completed a human trial using allogeneic SC for the treatment for heart disease. An intravenous drip was used to deliver of the shelf MSC to patients that had recently suffered a heart attack. No deaths occurred, and the treatment is now widely thought as safe [109].

Today there is no regulatory approved cell treatment for myocardial infarction, but research and clinical studies offer the hope for successful cell therapy in the next decades.

#### 2.2.2. Peripheral Arterial Disease

Lower limb ischemia causes a decreased blood flow in the lower leg with intense pain and swelling [114]. Recently, preliminary results of a Phase I clinical trial using adult SC treatment for severe limb ischemia was presented with endothelial progenitor cells (EPC) and MSC. The cells, obtained by bone marrow aspiration, were mixed and infused into damaged vessels. According to this study, there were no adverse effects as a result of the infusions. More importantly, their patients experienced a progressive and lasting improvement in clinical parameters including walking tests, oxygen pressure, angiography, and quality of life. The use of adult SC therapy in ischemia patients would allow the development of new mature and stable capillaries. These cells have shown the property of differentiation into endothelial or smooth muscle cells but also produce a significant amount of vascular growth factors [115–120].

*Harvest Technologies Corp*. (MA, USA) presented some positive results from a 30-patient clinical trial of a stem cell-based treatment of critical limb ischemia (CLI). Clinical evaluation of the patients with thromboangiitis obliterans disease conducted for 12 weeks showed that the treatment had significant clinical effect. The most important finding was that more than 85% of patients were able to save their legs. Other major endpoints also showed significant improvement including quality of life assessment and individual perception of pain; there was 100% reduction in the use of pain medications. Limb perfusion, as measured by TcPO2, also showed statistically significant improvement. Thirty-three percent of the patients had serious ulcers and 90% of these showed 90% or better wound closure in 26 weeks. There were no adverse events associated with the treatment.

In 2009* Pluristem Therapeutics Inc*. (Haifa, Israel) began a Phase I clinical trial with a placenta-derived SC product for the treatment of CLI (end-stage of peripheral artery disease). The different trials (12 patients) generally evaluated the safety of the product in patients with CLI.

#### 2.2.3. Ischemia Stroke

Cerebral infarct is a process, in which brain damage increases with time. Therefore the time when treatment is started is critical. At present, the only effective treatment (tissue plasminogen activator) has to be administrated very soon after the stroke [114]. In animal models, intravenous administration of hUCB cells to rats, after induction of stroke by occlusion of the middle cerebral artery, promoted the improvement of neurological function. The cells were mainly found in the cortex and the striatum of the damaged hemisphere and outside the brain, in bone marrow and spleen, and in very small amounts in muscle, heart, lungs, and liver. These authors found that some of the injected cells showed neuronal markers (NeuN2 and MAP2), astrocytic markers (GFAP), and endothelial cell markers (FVIII) [121].

Actually, IPS cells should be ideally generated without using viral vectors and without teratoma formation for being suitable for clinical use. But today the main clinical trials in topic use autologous bone marrow MSC [122–124].

#### 2.2.4. Nervous System and Neurodegenerative Diseases

The classical notion of a renewal of adult neuronal cells is today questioned, but the therapeutic applications still remain uncertain. The spinal cord repair is currently the purpose of a great deal of work after injury [125, 126] (10 trials) with bone marrow SC. In 2010, a first study from cells derived from embryo SC producing oligodendrocytes was carried out in a volunteer in connection with the* GERON Company *[127, 128]. So far, no information has been given on the result of the test and the study seems to have been stopped. In France, one group is involved in a clinical study, in cooperation with a German team, using autologous bone marrow SC. Other studies should also begin in USA, Portugal, and China.

Amyotrophic lateral sclerosis (ALS) is a neurodegenerative disease characterised by a progressive muscle weakness that can result in paralysis and death. The multicausality of neuron death poses a considerable problem to the development of new therapeutic strategies, including cell therapy. Numerous hypotheses have been developed about the origin of ALS, but it seems that the immune system may be involved. Cell transplantation approaches in ALS remain to generate a neuroprotective environment for degenerating motor neurons by transplantation of nonneuronal cells, rather than to replace lost motor neurons. Among the cell therapy approaches tested in motor neuron disease animal models, systemic injection of human cord blood mononuclear cells has proven to reproducibly increase the life span of SOD1G93A mice, a model of familial ALS, even if only few transplanted cells were found in the damaged areas. Bigini et al. showed that human cord blood (mononuclear cells) significantly enhanced symptoms progression and prolonged survival in SOD1G93A mice and were localized in the lateral ventricles, even 4 months after administration [129]. However, hCB-MNCs were not found in the spinal cord. These results strengthen the hypothesis that the beneficial role of transplanted cells is not due to cell replacement but is rather associated with the production and release of circulating protective factors. They observed in this study that hCB-MNCs release a series of cytokines and chemokines with anti-inflammatory properties that could be responsible of the functional improvement of mouse models of motor neuron degenerative disorders. The clinical trials (25 beginning of 2014) are mainly focused on Parkinson [130] and Alzheimer [131] diseases and amyotrophic lateral sclerosis [132] with bone marrow and umbilical cord SC.

#### 2.2.5. Bone and Cartilage [133–135]

The prevalence of osteoarthritis and degenerative joint disease will increase in the near future, driving the market for SC therapies for joints and cartilage [136]. In fact, most of the pioneering work using stem cells for bone and tendon repair was carried out in the veterinary field, mainly injuries in racehorses. Adult MSC are able to differentiate in bone, cartilage, tendon, ligament, and muscle. Today the most studied source for bone and cartilage is the bone marrow with different scaffolds. For bone the problem is more complex, when compared to cartilage, because bone is a vascularized tissue and the formation of mineralized bone matrix is not sufficient to lead to a functional tissue [137–139].

Cartilage is a mesenchymal tissue composed of one cell-type (chondrocytes), extracellular matrix (ECM), and water. Chondrocytes represent only 1-2% of cartilage volume. The cartilage ECM is composed of collagen fibers (mainly type II collagen) supporting glycoproteins and proteoglycans which have a protein core associated with glycosaminoglycan molecules such as hyaluronic acid (HA) and chondroitin sulfate. The tissue fluid, mainly water, contributes to the particular mechanical properties of cartilage and provides nutrition and exchange with synovial fluid and with extracellular fluid or other adjacent tissues. Hyaline cartilage functions with minimal friction. It demonstrates an excellent ability to provide and adaptation to compression and distributes loads on the surface of the joint [140, 141].

Because of the limited self-healing capacity of cartilage, repair of articular defects caused by degenerative joint diseases or traumatic injuries represents an open challenge and current therapies for cartilage repair are inadequate for restoring form and function [142–144].* In vitro* preparation of functionally developed biocartilage substitutes is an attractive concept for future clinical treatments of cartilage injuries and degeneration. Today a FDA approved cellular-based therapy for cartilage defects uses chondrocytes [145]. In this application, autologous cells are harvested from a biopsy and expanded* ex vivo* to obtain a large number of cells for transplantation. Autologous expanded chondrocytes have a low risk of immune rejection but they have a tendency to dedifferentiate (loss of phenotype)* in vitro*. In other words, the influence of mechanical forces on cell function* in vitro* has been demonstrated for engineering cartilage and bones [146]. In cartilage production, dynamic mechanical stresses on chondrocytes and MSC promote differentiation and increase matrix production [147–149].

Many clinical studies performed have demonstrated the therapeutic potentials of MSC from bone marrow or adipocytes [150–153]. Wharton's jelly MSC offer another source that has proven a chondrogenic differentiation potential and could be used in an allogenic context [46, 154–156].

While MSC therapy is promising, the incomplete understanding of their biological characteristics and function limits today the utilization of MSC in clinical application [157]. Role of growth factors, cytokines, receptors, transmembrane signalling, and adhesive proteins in MSC interactions are still elusive. In a study of the effects of MSC in a caprine model of traumatic osteoarthritis it was showed that intra-articular delivery of autologous cells to meniscectomized joints resulted in significant meniscal and regeneration and chondroprotection. In another study, subcutaneous implantation of hydroxyapatite scaffolds loaded with allogenic MSC allows cartilage obtention. The present clinical trials for cartilage repair are mainly focused on osteoarthritis 29 trials with auto- or allo-SC from bone marrow, umbilical cord, or adipose tissues [158–160].

#### 2.2.6. Dermatology

In mammals, cell renewal on the external surface of the skin is ensured by the keratinocytes of the basement layer which divide actively and are differentiated into cells of the stratum corneum. That activity implies the existence of SC. Unlike the SC of hair follicles confined in a niche, the SC of the* epidermidis* are spread along the basement membrane. The main clinical trials [10] are mainly on limb ischemia in diabetic patients [161, 162].

#### 2.2.7. Pancreas and Diabetes [163]

The cell graft appears to be an alternative to the medical treatments for pancreas diseases. The first attempt of cell therapy by grafting of islets of Langerhans was published more than ten years ago [164–167]. Other cell sources have also been proposed for pancreas and diabetes cell therapy like adult [168, 169] or fetal [170–172] MSC, embryonic stem cells [173–175], or even IPS [176, 177] because of their differentiation potential into insulin-producing cells and their immunomodulatory properties. Two studies with different therapeutic approaches are currently investigating the influence of cord blood stem cells on improving the function of pancreatic beta cells. In the first approach, children with young-onset diabetes are infused with autologous cord blood without chemotherapy. Initial results have shown that such autologous cord blood transplantation without chemotherapy has not resulted in adverse effects but has not significantly improved the situation either. All children are still dependent on administration of insulin. In the second approach, adult patients with newly diagnosed diabetes mellitus underwent nonmyeloablative chemotherapy after receiving reinfused stem cells from autologous bone marrow. Different trials on diabetes [22] type 1 or 2 are mainly performed with autologous or allogenic bone marrow or Wharton's jelly MSC [178–181].

#### 2.2.8. Liver Diseases

In response to a variety of chronic injuries such as hepatitis, alcohol or drug abuse, metabolic diseases, autoimmune attack of hepatocytes or the bile duct epithelium, and congenital abnormalities, liver fibrosis occurs and finally leads to hepatic cirrhosis and liver failure. Liver transplantation is the accepted treatment option for this end-stage liver diseases and acute liver failure resulting in irreversible liver dysfunction. However, it is limited by the shortage of donor organs. Moreover, it is difficult to accept such a heavy surgical treatment for some patients because of the shortage of donor organs. In fact, correction of hepatocyte functional deficiency is the prime goal of liver transplantation. There is growing evidence in support of cell therapy. As an alternative to liver transplantation, some authors tried to use hepatocytes to treat patients with liver diseases instead of liver transplantation. However, the obstacle against their clinical applications is the requirement of large number of hepatocytes that are not available from patients themselves and as well as from other donors either. Thus, it is necessary to search for a novel source of cells. SC therapy has been accepted as one of the new approaches to recolonize liver. Several studies reported the hepatocyte differentiation potential of embryonic, fetal, or adult MSC but also IPS [182–187].

As the liver contains three different cell types, which are organized in three-dimensional structures, growth and regeneration processes are highly complex. Therefore the idea of using one-type of SC leading to these three types of cells to repair liver is acceptable. Various populations of SC are under investigation in terms of their regenerative capabilities. Recently, studies showed that extrahepatic adult MSC of different origins have demonstrated their ability to express a hepatocyte-like phenotype after being differentiated* in vitro*. These cells which include MSC derived from bone marrow, umbilical cord, adipose tissue, and placenta are used in 32 trials mainly for cirrhosis (after hepatitis, alcohol abuse, and liver transplantation) [188–191].

#### 2.2.9. Urology and Erectile Dysfunction

The group of Atala in USA performed urethra transplant in young patients, prepared* in vitro* from bladder cells cultivated on a collagen and polyglycol acid matrix [192].

Recently, the SC therapy for erectile dysfunction has been investigated. Transplantation of SC (adipose-derived stem cells or bone marrow stem cells…) was performed by intracavernous injection [193, 194]. More recent studies used combinatory therapy by supplementing stem cells with angiogenic proteins. The different studies reported better erectile function after SC mainly by intracavernous injection [195].

The main potential applications are postprostatectomy and postradiotherapy, diabetes associated erectile dysfunction, and Peyronie's disease [196]. Human clinical trial of erectile dysfunction with SC is not yet approved as treatment but one clinical trial in Korea was published in 2010 and two preclinical trials have been approved in USA and France.

#### 2.2.10. Retina

The different ophthalmologic treatments with SC (mainly bone marrow) are related to retina diseases, macular degeneration, glaucoma, and hereditary dystrophy [197–199].

#### 2.2.11. Hematology: Preparation of Red Blood Cells

*In vivo* production of red blood cells (RBC) can be of a great practical interest. Recently, RBC preparation has been possible* in vitro* based on CD34+ stem cells [200, 201]. The protocol is in three steps: (1) proliferation and induction of the erythroid differentiation, (2) culture on a model reproducing the physiological microenvironment with mesenchymal cells, and (3) culture in the presence of stromal cells alone and without any growth factor. With this protocol, the cells undergo the various phases of differentiation to red cells. The industrial development would require developing suitable bioreactors. Another solution would be to have a permanent and unlimited supply of blood products. A first option is to use embryonic SC whose differentiation gives: first CD34+ stem cells, then erythrocytes [202]. Another approach consists in using induced pluripotent SC or IPS [203]. Different lineages of IPS and/or embryonic SC are currently used experimentally; beyond the difficulties in controlling complete differentiation, one major issue to be solved is that of insufficient yields [204].

### 2.3. Stem Cells and Whole Organ Engineering

The relevance of research into the creation of reconstructed organs is justified by the lack of organs available for transplantation and the growing needs for an ageing population. On a technical level, the development of these reconstructed organs involves two complementary stages: decellularization of the target organ with a need to maintain the structural integrity of the extracellular matrix and recellularization of the matrix with stem cells or resident cells [205, 206].

Whole organ engineering like liver, kidneys, heart, or lung is particularly difficult because of the structural complexity and heterogeneity of organ and cell types. But new ways of researches are currently focused on the matrix to support recellularization and a promising approach is the direct use of extracellular matrix of the whole organ. Thus rodent, porcine, and rhesus monkey organs have been decellularized to obtain a scaffold with preserved extracellular matrix and vascular network.

Decellularization can be achieved through an intra-arterial infusion of a solution containing the detergent Triton X-100 and ammonium hydroxide. This method causes all the cellular elements to disappear, leaving elements of the extracellular matrix and the vascular system. Other methods of decellularization have also been used, employing other chemicals, enzymes, or physical ways (ultrasounds) [207, 208].

Several types of cell can be considered for recellularization purposes: SC (embryonic, fetal, and adult SC) or the patient's autologous cells. SC probably represent the ideal source of material due to their ability to proliferate. Their use appears to be limited, nevertheless, by their allogenic nature, which could possibly trigger an immune response and consequent rejection, in addition to the risk with ESC of the formation of teratomas* in vivo*. Fetal cells conserve their ability to proliferate and are easily differentiated without running the risk of induction of teratomas* in vivo*. These obstacles could be removed in future by using nuclear transfer techniques from the patient's somatic cells (IPS). Finally, the stem or progenitor cells present in most organs are another source of cells that could be used for* in vitro* organogenesis. But, they often remain difficult to define, isolate, and grow in culture.

Furthermore, the type and number of cells to be used for recellularization vary depending on the organ to be reconstructed. Apparently, specific cells of the organ to be reconstructed are indispensable. Other types of cell, such as endothelial cells and fibroblasts, are also needed, since they promote the functional cell phenotype and contribute to the structural organization of tissue. The matrix of the vascular system of the organ to be reconstructed needs to be reendothelialized so as to orientate the blood flow and prevent thrombosis.

Currently, growing organs* in vitro* and* ex vivo* can take several weeks until they have completely developed in the matrix. For seeding the use of an extracorporeal pulsating or continuous infusion system (bioreactor) is indispensable for providing the cells with an oxygen supply and keeping the infusate at a constant temperature [209, 210]. The infusion liquids are derived from the culture media used for the cells in question. They need to contain growth factors or other molecules that are more specific to each organ. Finally, there is another hypothetical possibility for recellularization, the transplanting of a decellularized organ into the recipient, in the hope that recellularization will occur directly from the recipient's own cells.

Encouraging work has recently shown the feasibility of creating bioorgans for the reconstruction of heart, lungs, liver, and kidneys. Clinical applications still remain a distant prospect, however.

#### 2.3.1. Heart

Heart construction could be an alternate option for the treatment of cardiac insufficiency in the future. It is based on the use of an extra-cellular matrix coming from an animal's heart and seeded with cells likely to reconstruct a normal cardiac function. Though the decellularization techniques now seem to be under control, the issues posed by the selection of the cells capable of generating the various components of cardiac tissue are not settled yet. In addition, the recolonization of the matrix does not only depend on the phenotype of cells that are used but also impacted by the nature of biochemical signals emitted. The complexity of those problems results in the full replacement of the heart with a biomaterial substitutes to standard transplanting is one prospect [211]. However, it is more realistic to hope, in the medium run, partial replacements of the heart with recellularized matrices reinforcing portions of the failing myocardium or with direct cellular therapy with SC.

The decellularization of animal hearts (rats and, more recently, hearts of large mammals) has been performed by D. Taylor through the infusion of chemical detergents. This study shows that the integrity of the matrix (collagen, fibronectin, laminin, fiber orientation, etc.) can be maintained as well as the permeability of the vascular tree and the competence of the heart valves [212].

Recellularization is more difficult due to the diversity of the cell populations that need to be reconstituted. Three ways to achieve this goal can be considered. (i) Use of a single population of pluripotent cells is capable of giving rise to all types of heart cells through the effect of environmental signals (an approach that appears currently to be rather unrealistic). (ii) Use of adult cells already differentiated for target lineages. The obtaining of fibroblasts and vascular cells can be achieved, especially as they can be taken from a future “recipient” of the reconstituted organ, as has been successfully demonstrated in the creation of implantable blood vessels. (iii) The third intermediate strategy consists of using a single population of progenitor cells at the mesodermic stage that would be liable, depending on the signals produced by the host tissue, to achieve differentiation* in situ* in the three main cell types (cardiomyocytes, endothelial cells, and smooth muscle cells). The problem of obtaining cardiogenic cells is also more complex since they not only need intrinsically contractile properties, but they must also be capable of coupling and modulating their frequency in response to neurohumoral or pharmacological stimuli. The plasticity of adult somatic cells is limited; however, it does not allow them to differentiate into cardiomyocytes. This property is only possessed by pluripotent cells, capable of acquiring a cardiac phenotype under the influence of the appropriate signal inducers. Such pluripotent cells could be human embryonic stem cells (hESC) whose allogenic character poses the problem of rejection (to say nothing of the ethics debate) or autologous, adult somatic SC rendered pluripotent through reprogramming (IPS). Regardless of the origin of such pluripotent cells, however, their clinical use implies an* in vitro* differentiation stage and then a selection process so that only the cardiogenic progenitors would be used. More recently, a direct conversion of the adult cells (fibroblasts) into cardiomyocytes has been proposed, again passing through the pluripotent cell stage. This approach still seems to be remote for clinical applications [213].

An important challenge is the transfer of cells into the matrix to recolonized [214]. While cell infusion destined for the vascular system appears to be logical for the endothelium, intramural injection of cells for cardiogenic purposes is less obvious.

In summary, by the complete replacement of a human heart by another heart constituted from a matrix of animal origin and seeded by cells capable of providing the organ with effective, mechanical activity remains a remote prospect and is unlikely to become a reality within the next 10 to 20 years.

Another strategy for cardiac repair is the preparation of cardiac patch [215, 216]. The construction of the high biocompatible biomaterials pretreated with SC will offer a promising method to improve the effects of SC therapy for myocardial infarction. Thus the development of this cardiac SC patch has high therapeutic perspectives for the treatment of the disease and prevention of the chronic heart failure. However the materials suitable for the treatment of MI need to have specific quality: biocompatibility, resistant to the mechanical force* in situ*, suitable for the cell amplification, and being with suitable size of pores for the cell communication which is necessary for the formation of the functional tissue. Under microscope, the pore size needs to be at least 50 *μ*m which is necessary for the vascularization of the patch and assure the MSC metabolism. The biological materials have more advantages than artificial materials because the integration of the cells is optimal for the construction of the cardiac SC patch. As the MSC derived from Wharton's jelly are easy to collect, the umbilical artery can be collected at the same time. The natural matrix of the umbilical artery possesses the essential property for the construction of a biocompatible cardiac patch.

#### 2.3.2. Lungs

About fifty million people throughout the world are living with chronic respiratory failure at a terminal stage. The only treatment for this disease that seriously reduces life expectancy is, in selected cases, lung transplantation, but the results still are poor.

A tracheobronchial graft remains a challenge [217–221]. Research has not yet found an ideal cell substitute for the airways of the lung. Failures have been observed with synthetic prostheses, bioprostheses, tracheal allografts, and autografts. In fact, not only epithelial tissue regeneration but also even cartilaginous regeneration has been observed. Research seems to indicate that this regeneration of tracheal tissue might be possible from an aortic matrix and SC taken from bone marrow [217]. Studies have been performed in humans in the context of extended cancer of the trachea and conservation surgery in cases of lung cancer. The research has also contributed to better understand tissue regeneration mechanisms [222].

Pulmonary regeneration using SC is more complex [223]. In fact, several types of local progenitor cells that contribute to cell repair have been described at different levels of the respiratory tract. Moving towards the alveolus, one finds bronchioloalveolar SC as well as epithelial cells and pneumocytes. In the category of “local SC,” cells of the subpopulation have been identified that are differentiation markers which* in vitro* mimic stromal mesenchymal cells. The role of these cells in tissue repair has been demonstrated in animal models. Recently it was described that resident, multipotent pulmonary SC are capable of self-renewal as well as clonogenicity. The phenotype and functional characteristics of these new cells have been specified* in vitro* and* in vivo*.

The lung also contains resident specific MSC that have been described and characterised [224–226]. These cells do not play a direct part in epithelial renewal but establish communication with the epithelium, thus ensuring their role as a local cytoprotector [227].

Finally, numerous studies performed on animals have shown a beneficial role played by exogenous MSC produced by bone marrow. The effects observed in lesional pulmonary edema, sepsis, pulmonary hypertension, and even idiopathic pulmonary fibrosis have resulted in clinical applications that are currently being assessed [228–231]. The immunomodulatory, anti-inflammatory, antiapoptotic, and angiogenic properties of MSC today place these cells at the heart of tissue repair. Contrary to past hypotheses, these cells do not seem to differentiate themselves into alveolar epithelial cells and their method of action would involve paracrine mechanisms, not all of which have as yet been explained.

With respect to the creation of a bioartificial lung,recent works on the subject have been realized with decellularized rat lung in order to obtain a supporting matrix [232]. Epithelial and endothelial cells were then injected into a pulmonary matrix followed by a five-day incubation period in a bioreactor. Morphological studies found an aspect closely resembling the animal's own lung with respect to the alveolar cells (volume, number, and size) and* in vitro* physiological studies also showed that the ventilatory capacities and gas exchanges had also been maintained. An* in vivo* implantation of the bioartificial lung produced spontaneous ventilation for six hours. After this, pulmonary edema occurred. Several research routes, such as improvement in differentiation and maturation of the injected cells, a longer incubation period in the bioreactor and optimization of postoperative ventilation have been proposed.

Recently, others authors also decellularized a rat lung using chemical treatment, retaining only the framework matrix of the lung. This decellularized lung was then placed in a bioreactor that was used to mime the physiological conditions (negative pressure and pulsatile vascular perfusion). Epithelial cells from new-born rats were injected through the trachea and endothelial cells were injected into the vascular system. After four to eight days of incubation, this biolung was grafted on to a rat [221]. The compliance measurements were substantially different between the native lung, the decellularized lung, and the lung produced by bioengineering, with greater opening pressures reflecting a less functional surfactant in the bioengineered lung. Yet there was nothing to indicate rigidity of the matrix, thus ruling out the development of fibrosis, and gaseous exchanges were covered, attesting to the functional nature of this lung.

The decellularization of lungs was then reproduced on the lungs of pigs, nonhuman primates, and even humans [233–235]. Embryonic SC or MSC were used to cellularize decellularized lungs [235–237].

This initial research opens up a promising route for developing a functional bioartificial lung, with the prospect of application to humans within 15 to 20 years [238]. However many questions remain to be answered: is the use of a decellularized pulmonary matrix the only possible solution? Which cells should be chosen for recellularization, MSC or resident pulmonary cells? What is the optimal incubation time in a bioreactor? Would the technique be applicable to the human lung with its very extensive alveolar surface?

#### 2.3.3. Liver

Recent researches have shown that it was possible to use decellularized liver treated by detergents as scaffold, which keeps the entire network of blood vessels and the ECM [239]. The intact blood vessel network will mimic the circulation in organ and provide appropriate oxygen and nutrient supply for bioartificial liver [240]. The ECM is composed of a complex mixture of molecules and arranged in three-dimensional spatial organization that support the cell seeding, growth, and differentiation. Decellularized liver keeps the texture of the original organ. This natural structure can provide a three-dimensional matrix in favor of cell proliferation, differentiation, and function, which promotes the emergence of the idea to use decellularized organ in bioengineering liver. The liver decellularization is carried out by perfusing detergents like Triton X-100 or sodium dodecyl sulfate,* via *the portal vein [241]. This method can destroy cell membrane and take off debris of cells and at the same time keeps the extracellular matric complete with blood and biliary vessels. This matrix maintains the liver-specific proteins proportions for collagens I and IV, fibronectin, and laminin. The intact vascular system is useful for recellularization.

Besides decellularized whole organ scaffold, the choice of cells selected to repopulate a decellularized liver scaffold is critical to the function of bioengineered liver. At present, potential cell sources are hepatocyte and MSC. SC, such as liver stem cells, ESC, IPSCs, and MSC, are a promising alternative for primary hepatocytes. Recent studies have shown that MSC originated from extrahepatic tissues can differentiate into endoderm cell-lines as hepatocytes. Several methods have successfully differentiated MSC into hepatocytes, such as stimulation MSC by cytokines as growth factors direct and indirect coculture of MSC with hepatocytes, or promotion of MSC differentiation in a three-dimensional matrix. In some cases, differentiation of MSC into hepatocytes can also be an alternative approach for whole organ transplantation in treatment of acute and chronic liver diseases [183]. Moreover, it has been shown that decellularized liver scaffold supports hepatic differentiation of MSC, leading to cells with morphological and functional characteristics of mature hepatocytes [242, 243].

#### 2.3.4. Kidney [244–246]

The kidney is certainly one of the most difficult organs to reconstruct due to its tissue complexity and the heterogeneous nature of the cells from which it is constituted. There is relatively few researches on kidney autoconstruction, though experiments performed on rats, pigs, and monkeys [247]. The first demonstration of the feasibility of the technique was provided by Ross in the rat [248]. They seeded the decellularized organ with pluripotent murine embryonic stem cells antegrade through the artery or retrograde through the ureter. The cells introduced were differentiated into glomerular, tubular, and vascular structures. They nevertheless lost their embryonic phenotype as it could be seen from the appearance of immunohistochemical markers. Nakayama et al. [249] decellularized sections of the kidneys taken from macaques at various growth stages from fetus to adult,* via* intermediate ages, with the aim of optimizing decellularization techniques and recellularization* in vitro* [249, 250]. They demonstrated that the appearance of Pax-2 and vimentin markers after the cells had been implanted originated from the kidneys of the fetus.

As with other organs, the research into the construction of a kidney raises numerous questions about the preparation of a matrix and the sources of the cells used for recellularization. Biological matrices have proved their superiority over the synthetic matrices sometimes used in tissue engineering. In the case of the kidney, the most frequently employed matrices are allogenic, even though xenogeneic matrices can be considered, although they might be subject to specific immunological and regulatory issues.

In summary, a large number of questions and problems remain to be solved before a kidney can be prepared or constructed from ECM. Furthermore, none of the “self-constructed” organs in animals has proved to be capable of performing the vital function in the recipient for longer than a few hours. In the case of the kidney, no transplant has yet been reported even though it is the main challenge for research. The objective remains plausible, however, even if clinical applications appear to be very remote, certainly not before 15 to 20 years.

## 3. MSC Secretome for Tissue Repair: Towards a Cell-Free Therapy

Even if initially MSC were proposed for cell therapy based on their differentiation potential, the lack of correlation between functional improvement and cell engraftment or differentiation at the site of injury has led to the proposal that MSC exert their effects not through their differentiation potential but through their secreted product [251, 252]. The secretion of bioactive factors is then thought to play a predominant role in the mechanisms of action of MSC. Haynesworth et al. [253] were the first to report that MSC synthesize and secrete a broad spectrum of growth factors, chemokines, and cytokines that could exert significant effects on cells in their vicinity. Since that, many researches have been focused on the characterization of the MSC secretome, including both soluble factors and factors released in extracellular vesicles (e.g., exosomes and microvesicles) and their therapeutic potential [254–256].

The results from most investigations show that MSC-conditioned medium or its components mediate some biological functions of MSC. Several studies have reported that MSC-derived exosomes have functions similar to those of MSC, such as repairing tissue damage, suppressing inflammatory responses, modulating the immune system, or even decreasing cancer cells proliferation [257–264].

Together these studies provided pivotal support for the paracrine hypothesis such that MSC therapy is increasingly rationalized on MSC secretion rather than its differentiation potential. However, the mechanisms are still not fully understood and the results remain controversial. Compared with cells, exosomes are more stable and reservable, have no risk of aneuploidy, a lower possibility of immune rejection following* in vivo* allogeneic administration, and may provide an alternative therapy for various diseases.

## 4. Conclusions

The regeneration of tissues and organs and the use of SC for clinical uses are and will remain a challenge for the development of cell therapy and tissue engineering. Fetal and adult SC and in particular MSC provide exciting therapeutic tools of regenerative medicine. However basic research should be developed to better understand the biological process and molecular mechanism of SC differentiation, as well as the role of the mechanical signals.

Several challenges should be overcome:

- increase of the yield of preparation of the differentiated stem cells and study of the heterogeneous character of the preparations;
- possibility to have a standardized and reproducible product (preparation of controlled batches);
- technical problems regarding the definition of scaffolds, cells used, long-term stability, and culture medium. In particular, the impact of the biomaterial used remains to be defined;
- grafting (biotissue can be introduced via direct cell implantation (cell therapy), biotissue transplantation, or gene therapy);
- risk of teratogenic effect and of immune reaction (i.e., in the umbilical cord cells the immune risk being weaker);
- religious and legal issues with respect to the different country regulations.

Current knowledge allows optimism for the future but definitive answers can only be given after long-term randomized and controlled clinical trials.
